# Lactase persistence, NOD2 status and *Mycobacterium avium* subsp. *paratuberculosis* infection associations to Inflammatory Bowel Disease

**DOI:** 10.1186/1757-4749-4-6

**Published:** 2012-06-28

**Authors:** Natalia Elguezabal, Susana Chamorro, Elena Molina, Joseba M Garrido, Ander Izeta, Luis Rodrigo, Ramón A Juste

**Affiliations:** 1 Animal Health Department, NEIKER-Instituto Vasco de Investigación y Desarrollo Agrario, Berreaga, Derio, Bizkaia, 1.48160, Spain; 2 Instituto Biodonostia, Hospital Donostia, Paseo Dr Beguiristain 115, San Sebastián, 20014, Spain; 3 Servicio de Aparato Digestivo, Hospital Universitario Central de Asturias, C/ Celestino Villamil, Oviedo, s/n. 33006, Spain

**Keywords:** *Mycobacterium avium* subspecies *paratuberculosis*, Inflammatory bowel disease, Crohn’s disease, Ulcerative colitis, Lactase persistence, NOD2, C/T_−13910_ genotype

## Abstract

**Background:**

Inflammatory Bowel Disease (IBD), which includes both Crohn’s disease (CD) and ulcerative colitis (UC), is caused by a complex interplay involving genetic predisposition, environmental factors and an infectious agent. *Mycobacterium avium* subsp. *paratuberculosis* (MAP) is a promising pathogen candidate since it produces a chronic intestinal inflammatory disease in ruminants that resembles CD in humans. MAP is a ubiquitous microorganism, although its presence in the food chain, especially in milk from infected animals, is what made us think that there could be an association between lactase persistence (LP) and IBD. The LCT mutation has brought adaptation to dairy farming which in turn would have increased exposure of the population to infection by MAP. NOD2 gene mutations are highly associated to CD.

**Methods:**

In our study, CD and UC patients and controls from the North of Spain were genotyped for the lactase gene (LCT) and for three NOD-2 variants, R702W, G908R and Cins1007fs. MAP PCR was carried out in order to assess MAP infection status and these results were correlated with LCT and NOD2 genotypes.

**Results:**

As for LP, no association was found with IBD, although UC patients were less likely to present the T/T_−13910_ variant compared to controls, showing a higher C-allele frequency and a tendency to lactase non-persistence (LNP). NOD2 mutations were associated to CD being the per-allele risk higher for the Cins1007fs variant. MAP infection was more extended among the healthy controls (45.2%) compared to CD patients (21.38%) and UC patients (19.04%) and this was attributed to therapy. The Asturian CD cohort presented higher levels of MAP prevalence (38.6%) compared to the Basque CD cohort (15.5%), differences also attributed to therapy. No interaction was found between MAP infection and LCT or NOD2 status.

**Conclusions:**

We conclude that LP is not significantly associated with IBD, but that MAP infection and NOD2 do show not mutually interacting associations with IBD.

## Background

Inflammatory bowel disease (IBD) is a pathological enteritis characterized by chronic regional inflammatory infiltrate of the intestinal wall and associated lymph nodes that comprises Crohn’s disease (CD) and ulcerative colitis (UC). Etiology of IBD remains unclear, although inflammation can be a result of inappropiate chronic activation of the innate and adaptive mucosal immune systems in individuals with genetic modifications [1]. Exposure to pathogens seems to be a potential cofactor for disease development [2] meaning that the disease could be induced by an infectious agent in genetically susceptible individuals. *Mycobacterium avium* subsp. *paratuberculosis* (MAP) is a probable pathogen candidate for at least one subtype of IBD, CD, since it is responsible for a disease in ruminants of similar clinical and histological conditions named Johne’s disease (JD) or paratuberculosis [3-5]. The connection between both intestinal inflammatory bowel diseases, human and ruminant, was first described in the early 1900s [6,7].

Although the possible link between MAP and CD remains controversial, improvements in isolation [8,9] and genetic techniques [10-14] are providing evidence that MAP might play a causative role in the development of CD along with genetic [15] and immunological factors [16]. Mutations in the NOD2 locus are highly associated with CD in Europeans [17] and a recent genome-wide study from China [18,19] has shown that a high proportion of leprosy patients have many of the same genetic mutations found in patients with CD including NOD2/CARD15 mutation. All these findings lend support to the mycobacterial etiological hypothesis in CD.

Lactase deficiency has been commonly found in adults with IBD, mainly in CD patients. In fact, lactase non-persistence (LNP) was thought to be a predisposing cofactor that could activate the disease. Some studies have shown that the prevalence of LNP is greater in IBD patients compared to controls [20-22] and more common in patients with CD (40-46%) when compared to UC (13-16%) [20,22].

Previously [23], we raised the hypotheses that a correlation between LNP and CD incidence could support the idea that MAP is the causative agent of CD. Lactase persistence (LP) dominant mutation could have been originated as a consequence of adaptation to dairy-farming and exposing humans to MAP through milk consumption and overall close contact with infected cattle. In our observational epidemiological study higher CD incidence correlated with lower LNP frequency, this is higher LP frequency. Similar conclusions were drafted by a recent meta-analysis that studied the impact of lactose on LP and IBD, among other diseases [24].

When assessing previous studies we find that the relation between CD and LNP is also questionable [25-27]. Buning *et al.*[28] genotyped for the lactase gene (LCT) which encodes for the lactose phlorizin hydrolase (LPH) that splits lactose in the small intestine and failed to find an association between the C/C_−13910_ and G/G_−22018_ genotypes, accepted markers for LNP, with susceptibility to CD and UC. However, Nolan et al [29] showed an association between LP and the risk of CD in New Zealand.

Since, both UC and CD are thought to be multifactorial disorders where polygenic dysfunction could be the ground for inflammatory changes triggered by MAP. The aim of the present work was to study the possible interplay of genetic susceptibility for LNP (LCT status) and CD (NOD2 status) and the presence of MAP among IBD patients and controls from the North of Spain that would support an etiological role of MAP.

## Results

Complete data for MAP DNA presence in blood and both LCT and NOD-2 genotyping was accomplished for 278 subjects with IBD (173 with CD and 105 with UC) and 188 healthy controls. Demographic data, etiology of disease and therapy of these 466 individuals is shown on Table 1. Equal number of women and men were recruited and no significant differences in gender were found among groups. UC individuals were older than CD patients (p < 0.0001) and healthy controls (p = 0.002), probably being due to subject enrollment.

**Table 1:** Demographic information and etiology of Inflammatory Bowel Disease patients and controls

	**CD**	**UC**	**HC**
	**(n = 173)**	**(n = 105)**	**(n = 188)**
Gender (%female)	50,86	53,33	53,15
Age (yr)			
Mean	38,4+/−12,2	44,9+/−12,9	40,0+/−12,94
Range	16–77	21–69	19–61
ND	16,5 %	8 %	
Drugs (%)			
Azathioprine	43,35	15,20	-
Budenoside	3,47	4,80	-
Infliximab	15,60	2,90	-
Mesasalazine	57,23	63,80	-
Metronidazol	4,62	2,90	-
Prednisolone	8,67	11,40	-
Disease Location (%)			
Rectum (E1)	-	41,90	-
Left colon (E2)	-	17,10	-
Colon (E3, L2)	12,70	31,40	-
Ileum (L1)	42,2	-	-
Ileocolon (L3)	30,60	-	-
Upper digestive tract (L4)	2,90	-	-
ND	11,60	9,50	-
Active Disease (%)	16,18	14,28	-

CD: Crohn’s Disease, UC: Ulcerative colitis, HC: Healthy Controls, ND: Not determined.

The LCT genotypes for all subjects were analyzed and our results revealed a frequency of 21.2% for the LNP C/C_−13910_ genotype. LCT genotype distribution followed the Hardy-Weinberg principle. The frequencies of C/T_−13910_ alleles and genotypes stratified as a function of IBD type are presented in Table 2. No significant differences were detected on the percentage of C/C_−13910_ variants among UC and CD patients (25.7 and 21.4%, respectively) and healthy controls (18.6%). However, remarkably lower presence of the T/T_−13910_ variant was observed among UC patients (22.9%) compared to controls (38.3%) (p = 0.0075). The frequency of the T/T_−13910_ variant tended to be higher when UC patients and CD patients were compared (p = 0.1005). UC patients present a higher C allele frequency (51.4%) compared to healthy controls (40.2%) (p = 0.0091) and CD patients (45.5%) (p = 0.1775).

**Table 2:** **The distribution of LP/LNP genotypes and allele frequencies in patients with inflammatory bowel disease (IBD), Crohn’s disease (CD) ulcerative colitis (UC) and in controls (HC) [*n*(%)]**

				**Allele**	
		**Genotype**		**Frequency %**	
**Group**	**CC-13910**^1*^	**CT-13910**^**2**^	**TT-131910**^**2**^	**C**	**T**
**IBD (*n*** **= 278)**	64 (23.0)	138 (49.6)	76 (24.4)^a^	47.8	52.2
**CD (*n*** **= 173)**	37 (21.4)	84 (48.6)	52 (30)	45.5	54.5
**UC (*n*** **= 105)**	27 (25.7)	54 (51.4)	24 (22.9) ^b^	51.4	48.6
**HC (*n*** **= 188)**	35 (18.6)	81 (43.1)	72 (38.3)	40.2	59.8

^1^Lactase non-persistent genotype (LNP); ^2^Lactase persistent genotype (LP) *No significant differences were detected comparing LNP and LP genotypes between UC patients and CD patients (*P* = 0.409), CD patients and controls (*P* = 0.506) or IBD with controls (*P* = 0.255). Nearly significant differences comparing UC patients and controls (*P* = 0.154). Significant differences were detected when comparing TT genotype, ^a^IBD and controls (*P* = 0.013) and ^b^UC patients compared to controls (*P* = 0.0075). Nearly significant difference when comparing CD patients and controls (*P* = 0.1005).

NOD2 allele and genotype frequencies are summarized in Table 3. NOD2 mutation prevalence among CD, UC and HC were 23.69%, 8.51% and 12.76%, respectively. Homocygotes for R702W were only found in IBD patients and 1007 fs mutation rate was significantly higher in CD patients compared to UC (p = 0.021) and controls (p = 0.0135). Double mutants were only present in IBD patients, 9 (5,20%) in CD and 1 (0,95%) in UC patients. 1007 fs mutation was associated to upper digestive tract (OR, 8.37; 95% 1.26-55.45, P = 0.01) and in less extent to ileum (OR, 2.74; 95% 0.81-9.32, P = 0.095) in CD patients, whereas no association was found in the UC cohort.

**Table 3:** **The distribution of NOD2 genotypes in patients with inflammatory bowel disease (IBD), Crohn’s disease (CD), ulcerative colitis (UC) and in controls (HC) [*n*(%)]**

**NOD2 genotype frequency n (%)**			
**Group**	**WT/WT**	**Heterocygotes**	**Homocygotes**
**IBD (*n*** **= 278)**			
R702W	241 (86.7)	34 (12.2)	3 (1.1)
G908R	273 (98.2)	5 (1.8)	0 (0.0)
1007 fs	263 (94.6)	15 (5.4)	0 (0.0)
**CD (*n*** **= 173)**			
R702W	145 (83.8)	26 (15.0)	2 (1.2)
G908R	168 (97.1)	5 (2.9)	0 (0.0)
1007 fs	158 (91.3)	15 (8.7)*	0 (0.0)
**UC (*n*** **= 105)**			
R702W	96 (91.4)	8 (7.6)	1 (1.0)
G908R	105 (100.0)	0 (0.0)	0 (0.0)
1007 fs	105 (100.0)	0 (0.0)	0 (0.0)
**HC (*n*** **= 188)**			
R702W	171 (91.0)	17 (9.0)	0 (0.0)
G908R	186 (98.9)	2 (1.1)	0 (0.0)
1007 fs	183 (97.3)	5 (2.7)	0 (0.0)

WT: Wild-type

^*^ 1007 fs allele frequency, CD versus UC (p = 0.021) and CD versus controls (p = 0.0135).

MAP presence detected by PCR was more frequent in healthy controls (45.2%) than in CD or UC patients (21.38 and 19.04%, respectively). We next compared whether the frequency of MAP detection differed depending on the particular disease characteristics of patients. The affected area of the digestive tract did not correlate with MAP PCR result. No interaction was found when disease activity and MAP PCR result were compared in the CD group (OR, 0.768; 95% 0.27-2.18 p = 0.619). However, a suggestion of interaction was observed in the UC group (OR, 2.5; 95% 0.75-8.4 p = 0.128), meaning that MAP was detected more frequently in patients with active disease at sampling period in this cohort. Because therapy consisted in a combination of drugs in most cases, these could not be analyzed individually. No association was found when MAP presence was compared to drug combinations. Only when azathioprine was administered along with other drugs for CD therapy an interaction was found with MAP DNA in blood (OR, 2.29; 95% 1.09-4.82, P = 0.026), meaning that azathioprine combined with other drugs would not be effective in achieving MAP clearance.

When comparing MAP status with analyzed SNPs for LCT and NOD2 no association was found. More MAP infected individuals among the T/T_−13910_ genotype subjects (33.78%) were detected as compared to the C/C_−13910_ (28.28%) or C/T_−13910_ carriers (29.22%), although differences were not significant. Distribution of MAP PCR results within mutations and among cohorts is presented on Table 4.

**Table 4:** **NOD2 and LCT mutation distribution in MAP infected and non-infected individuals among Crohn’s disease (CD), ulcerative colitis (UC) and in control (HC) cohorts [*n*(%)]**

**Number of mutations**	**CD (n = 173)**		**UC (n = 105)**		**HC (n = 188)**	
	**MAP +**	**MAP -**	**MAP +**	**MAP -**	**MAP +**	**MAP -**
**NOD2**						
**0**	29 (16.76)	103 (59.54)	19 (18.10)	77 (40.96)	77 (40.96)	87 (46.28)
**1**	7 (4.05)	26 (15.03)	1 (0.95)	7 (6.67)	8 (4.26)	16 (8.51)
**2**	1 (0.58)	7 (4.05)	0 (0.00)	1 (0.95)	0 (0.0)	0 (0.00)
**LCT**						
**0**	27 (15.61)	109 (63.01)	14 (13.33)	64 (60.95)	73 (38.83)	80 (42.55)
**1**	10 (5.78)	27 (15.61)	6 (5.71)	21 (20.00)	12 (6.38)	23 (12.23)

No significant differences were detected among groups except for CD versus HC NOD2 0 and CD versus HC LCT 0 (p < 0.0001).

Subgroup analyses considering the Basque and Asturian CD patient cohort separately revealed no significant differences except for MAP infection as shown on Table 5. In this case, 38.6% of the Asturian CD patients were MAP PCR positive compared to 15.5% of the Basque CD subjects (p = 0.0015). Since differences in drug therapy could be a playing a role, we next analyzed the number of patients taking each drug and their MAP PCR result in both subgroups. Azathioprine showed to be less effective in Asturian patients (p < 0.0001), whereas effectiveness of Infliximab and prednisone was not significantly different among CD patients in both regions (p = 0.0738 for both).

**Table 5:** **Frequencies of LCT genotypes, NOD2 mutant carriers and mutations, and MAP PCR in the subgroups of CD patients [*n*(%)]**

	**Asturias**	**Basque country**	***P***
	**(n = 44)**	**(n = 129)**	
LCT genotype			
CC	8 (18.2)	29 (22.5)	0.5490
CT	19 (43.2)	65 (50.4)	0.4104
TT	17 (38.6)	35 (27.1)	0.1525
NOD2 mutants	8 (18.2)	33 (25.6)	0.3204
R702W	6 (13.6)	20 (15.5)	0.7610
G908R	0 (0.0)	5 (3.9)	0.1855
1007 fs	2 (4.5)	13 (10.1)	0.2561
MAP PCR positive	17 (38.6)	20 (15.5)	0.0015

The multivariate logistic regression analysis for IBD, CD and UC is summarized on Table 6. Testing for significant interactions between MAP infection, C/T_−13910_ genotype variants and presence of NOD-2 mutations for each group was performed. CD patients were less likely to be infected with MAP compared to controls (OR = 0.33, 95% CI, 0.21-0.53) and the likelihood of CD patients of being C/C_−13910_ or C/T_−13910_ carriers was similar to that of controls (OR = 1.44, 95% CI, 0.93-2.24) although a slight tendency was observed. For UC the trends regarding MAP infection were almost the same, i.e. less likely to be infected by MAP as compared to controls (OR = 0.29 95% CI, 0.16-0.50). However, among UC patients, it was much more likely to find a C allele carrier, i.e. a C/C_−13910_ or C/T_−13910_ genotype (OR = 2.16, 95% CI. 1.25-3.71). The model shows that MAP infection detection by blood PCR is less common in IBD patients compared to controls, not being predictive of disease. T/T_−13910_ genotype is less common in UC patients. It was more likely to find NOD-2 mutation carriers among CD patients.

**Table 6:** Multivariate logistic regression analysis of risk factors for subjects with inflammatory bowel disease (IBD), Crohn’s disease (CD) or Ulcerative Colitis (UC) versus those of healthy controls

	**Odds ratio (95% CI)**	***P***
**CD (n = 173)**		
C allele carrier in LCT	1.44 (0.93-2.24)	0.1
MAP (PCR positive vs PCR negative)	0.33 (0.21-0.53)	<0.0001
NOD-2 mutant	2.05 (1.18-3.58)	0.011
**UC (n = 105)**		
C allele carrier in LCT	2.16 (1.25-3.71)	0.005
MAP (PCR positive vs PCR negative)	0.29 (0.16-0.50)	<0.0001
NOD-2 mutant	0.67 (0.30-1.51)	0.338
**IBD (n = 278)**		
C allele carrier in LCT	1.65 (1.11-2.45)	0.013
MAP (PCR positive vs PCR negative)	0.31 (0.21-0.47)	<0.0001
NOD-2 mutant	1.74 (1.00-3.01)	0.046

In this analysis C allele carrier and MAP presence were treated as categorical values. Significance was considered when *P* < 0.05.

## Discussion

IBD and particularly CD are now thought to be the outcome of a complex synergism produced by predisposing genetic and environmental factors along with an infectious agent or shift in normal bacterial flora. The similarities that have been outlined between CD in humans and JD in both domestic and wildlife animals force us to look into mycobacteria, with a special focus on MAP. The main goal of this study was to indirectly demonstrate the mycobacterial role in CD by finding a correlation between LP and CD. This theory can be explained as a result of substantial milk consumption and the increased dairy ruminant farming associated to the intake of viable MAP both from milk and meat, and from environmental contaminated food in the LP adapted population. Also a possible interplay between the accepted susceptibility marker for CD, NOD2 and MAP status was sought. Our initial hypotheses have not been confirmed since we failed to find a significant association of LP with IBD or MAP infection, meaning that such correlation does not exist, at least in our study population. However, we can contribute with other interesting observations that will be further discussed.

We found that the frequency for LNP (C/C_−13910_ variants) in the North of Spain was 21.2%. This result agrees with previous studies for other European populations [30-32] where the prevalence of LNP in the general population was 15-25%. However studies conducted with subjects from other parts of Spain render LNP prevalences of 40.1% [33], 39.6% [34] and 34.6% [35]. The Basque Country and Asturias are historically milk producing regions. The climate in these areas presents less insolation compared to other parts of Spain and milk consumption might be an advantage for calcium absorption [32]. In this sense, higher T allele frequencies could be explained as the result of a certain biological advantage conferred by higher levels of milk consumption in adult life as a result of animal domestication and culturally transmitted practice of dairying [36].

Although other studies have assessed other SNPs [28], in our case, we only looked for the C/T_−13910_ variants since SNP G/A_−22018_ is thought to be in a linkage disequilibrium with the SNP C/T_−13910_[27].

When LCT genotypes were analyzed among groups, a significant lower frequency of the T/T_−13910_ genotype was found in UC patients. We are not sure about the meaning of this finding. In any case, if the C → T switch can be considered an adaptation, and T is dominant, an individual with T/T_−13910_ genotype would guarantee the transfer of adaptation to its progeny. Less T/T_−13910_ genotype individuals among UC patients would finally result in an increase in C/C_−13910_ and C/T_−13910_ progeny leading to a boost of LNP among UC patients. Some authors have suggested that UC have ethnically dependent increased rates of lactose intolerance [20-22], while other studies report that symptoms of milk protein allergy resemble UC consequently representing a subtype of the disease [37,38]. Our results show a tendency that supports both ideas.

Recently, Nolan et al [29] have reported that the T allele encoding LP was associated with an increased risk of CD in New Zealand. This discovery is in accordance with our initial hypothesis and with a previous study run by our group [39]. In that case a smaller population was studied and T allele frequency was higher in CD patients (61.9%) than controls (47.1%). Other studies have failed to detect significant differences when comparing the LCT genotype variants between CD or UC patients and controls [28]. Our present study shows similar T allele frequencies among cohorts and only marginally significant differences (p = 0.065) have been observed between the UC and HC groups. The discrepancy in results among studies could be due to sample size or to real differences between populations, either genetic or environmental, or both. In any case, our findings could lead to a hypothetical polymorphism that would be responsible for the different pathology of CD and UC, although we cannot discard that the association is simply accidental.

The NOD2 (nucleotide-binding oligomerization domain containing 2) protein is a receptor that interacts with muramyl dipeptide participating in the recognition of bacterial peptidoglycans in general [40]. Mycobacteria can therefore be recognized by this receptor [41] and a lost of function in NOD2 may in consequence lead to a decreased ability in keeping mycobacterial infections under control. We have assessed three independent mutations within the NOD2 gene [42] that are accepted to be associated to CD in Europeans, in order to assess NOD2 status among two populations in the North of Spain and also to relate it to MAP detection, since we hypothesize that subjects with a defective NOD2 will be infected with MAP.

NOD2 prevalence in CD patients was 23.69%, similar to that observed in other studies carried out in Europeans [43,44]. A higher number of individuals were found to present mutations in the CD group and double mutants were only detected in the IBD group, as expected. However, if we look at the different SNPs separately, only 1007 fs mutation carriers were significantly more abundant in the CD pool as compared to the UC and HC groups. Results presented in a report that summarizes geographic differences in the Spanish population concerning NOD2 support these findings [45]. In this work diversity in the SNP distribution for the NOD2 gene in different Spanish regions is described. The most remarkable result is that in the Galician and Asturian samples, NOD2 mutations do not always correlate with CD, since significant differences are not recognized when compared to the controls. This was the case for SNPs 8 (R702W) and 12 (G908R). This can be partially expected since SNP13 (1007 fs) is a loss of function mutation that should definitely correlate better with disease. In our present study, samples from CD patients from Asturias have been included along with samples from patients and controls from the Basque Country. The similarity between Asturias, Galicia and the Basque Country is that all regions are located in the Northern most part of Spain. These results may mean that the entire population in the North of Spain carries higher NOD2 mutation frequencies but not all of the individuals have developed or have been diagnosed CD. In this case, environmental factors should be considered.

Although NOD2 genetic variation has been related to ileal CD [43,46], we did not find clear associations between mutations and disease type in CD or UC patients, except for mutation 1007 fs which was more abundant in CD patients with upper digestive tract and ileal affections.

A great effort has been made to demonstrate the connection between MAP infection and IBD. MAP DNA detection has been found to be highly predictive of CD in some studies [10,47]. This was not so in previous studies carried out by our group, where we found that DNA from MAP was detected in a higher number of healthy individuals compared to IBD patients [12,13]. Once again, results presented in this report show that MAP infection is more extended among healthy individuals compared to CD or UC patients in the North of Spain. Frequent detection of MAP DNA in blood of humans may be due to the wide distribution of MAP in the environment and its presence in the food chain. MAP‘s recent isolation from meat products [48-50] and its presence in milk [51], makes these part of a transmission route that goes direct to human beings if these are not cooked appropriately [52]. The observation of higher frequencies of MAP in healthy controls than in patients is controversial, but it is compatible with a slow infection model of pathogenesis where healthy carriers that do not develop the disease are a larger proportion of the population than that showing clinical signs. Lower rates among clinical cases could be explained as the result of a reduction of bacterial burden by the antibiotic effect of standard IBD therapy [12]. Another hypothesis that could explain the IS900 PCR rates would be the presence of “protective strains” in Northern Spain. In this case, the entire population would be exposed to these strains. The group of subjects that mounts an appropriate immune response would benefit from infection, whereas genetically susceptible individuals with immune disregulation and/or dysbiosis would not be immunized correctly and would develop disease after infection with a non-protective strain. These diseased subjects would be administered therapy that would partially eliminate MAP and for this reason less MAP DNA would be detected among IBD patients. This hypothesis is less likely because it is more complex since it requires, in addition to the treatment effect, the existence a new type of MAP, the “protective” strains, that had never before been postulated. It is also possible that the IS900 PCR on peripheral blood might not be as efficient in detecting viable MAP (in patients) as in detecting non-viable MAP (in individuals controlling the infection). The difficulty with most of these explanations is that they are not specific of the populations we have studied and the control/patient ratio inversion should have been detected in other studies.

When subgroups were analyzed separately there were more MAP PCR positive CD patients in Asturias than in the Basque Country. Accordingly with the first hypothesis, we think that different response to therapies can be influencing this result.

The rationale for the present study was that there would be a correlation of MAP presence, LNP and NOD2 mutant carriage. This is the first work that intends to find this link. Other research groups have also failed to find interaction between NOD2 status and MAP presence in IBD [53]. In that case, the method used for addressing MAP infection was serological and we thought that MAP PCR could be more sensitive.

## Conclusions

Although previous studies have suggested an association between LNP and IBD at a population level, our study failed to find an association between the C/C_−13910_ genotype and IBD at an individual level. From these results we conclude that there is not a direct correlation between IBD and LNP although the C allele is more frequent in UC patients and this could be translated as a tendency to LNP among these patients. MAP infection is widely spread among the general population although it is not associated to LNP or to NOD2 status and it is more common in healthy individuals not under conventional IBD therapy. As for NOD2, this is the first study to our knowledge where NOD2 status has been assessed in a Basque population and as expected more mutant carriers were detected among CD patients in the whole.

Future studies should address a larger sample of patients and controls from different geographical regions within the same country and measure possible environmental aspects of each region as well as culture and dietary habits. Our results do not support a conventional explanation for a mycobacterial etiology of IBD, even taking into account the genetic markers included in this study. However, these results seem to confirm an unexpected association that needs to be clarified in future studies. In this sense, the current opinion that IBD has a genetic origin triggered by an external agent still is the most plausible. Demonstration that this agent is or is not MAP would require the improvement of detection tools, the selection of the correct specimens and the monitoring of the presence of the microorganism through a period of time, as well as looking into genetic factors.

## Methods

### Study population

Using a case–control design, IBD patients (*n* = 278) were recruited from three hospitals from the Basque Country: The Quirón Donostia Clinic in Gipuzkoa, the Hospital de Txagorritxu in Araba , and the Hospital de Galdakao in Bizkaia, and one hospital from the Principado de Asturias, Hospital Universitario Central de Asturias in Oviedo. Non-IBD patients designated as healthy controls (*n* = 188) were recruited from the Basque Country Blood Bank. A signed informed consent was obtained from all patients and controls who participated in the study.

### Sample collection and DNA extraction

Whole blood samples were obtained from each subject. All blood samples were coded to conceal the patient's identity and diagnosis to laboratory workers. All samples were processed within 4 hours after extraction in a class II bio-safety cabinet.

Genomic DNA was extracted from buffy coat cells as described previously [12] and it was used both for MAP IS900 nested PCR and genotyping. Briefly, one volume blood was incubated with one volume 155 mM ammonium chloride for 20 minutes to lyse the red blood cells. The tube was centrifuged (10 min 200 × g), the cell pellet washed twice with PBS and recentrifuged (10 min 200 × g). DNA was extracted and purified (QIAamp DNA Blood Mini Kit (QIAGEN GmbH, Hilden, Germany) and stored at −20°C until further use.

### Genotyping

#### LCT

Genotyping of the C/T_−13910_ (rs4988235) SNP was carried out by PCR–RFLP as described previously [54]. Briefly, genomic DNA went through PCR-RFLP using primers LCT-mod (5’-GCA ATA CAG ATA AGA TAA TGG AG-3’) and LCT-rev (5’-CCT CGT TAA TAC CCA CTG AC-3’). The PCR was carried out for 30 cycles of 94°C for 10 s, 52°C for 10 s, and 72°C for 10s. The amplification product (137 bp) was digested with NlaIV (GGN^NCC recognition site) 1U/reaction for 3 h at 37°C. When C was present in the polymorphic position, NlaIV digestion generated two fragments (22 and 115 bp). Digestion products were separated by 3% agarose gels electrophoresis and visualized after staining with gel red (Biotium).

#### NOD2

Genotyping of the CARD15/NOD2 gene was carried out by PCR–RFLP as described by Heliö [55]. Briefly, each NOD2 variant was assayed using initial amplification of the DNA sample by polymerase chain reaction (PCR) and subsequent analysis of the PCR products by restriction enzyme cleavage and gel electrophoresis on 12% polyacrylamide (R702W) or 3% agarose (G908R and 1007 fs).

Detection of the R702W (Arg702Trp:SNP8, rs2066844) mutation was done with forward primer, 5′-AGATCACAGCAGCCTTCCTG-3′ and reverse primer, 5′- CACGCTCTTGGCCTCACC-3′. The PCR product (185 bp in size) was digested at 37°C for 16 hours with 2 U of MspI, resulting in the following fragments: 20, 35, 54, and 76 bp in R702R homozygotes; 20, 35, 54, 76, and 130 bp in R702W heterozygotes; and 20, 35, and 130 bp in W702W homozygotes.

For assay of the G908R (Gly908Arg: SNP12, rs2066845) mutation, 5′- CTCTTTTGGCCTTTTCAGATTCTG-3′ was used as the forward primer and 5′-CAGCTCCTCCCTCTTCACCT-3′ as the reverse primer. The PCR product size from these primers is 163 bp. After digestion for 16 hours at 37°C with 2 U of Hha I, the following fragments were obtained: 163 bp in G908G homozygotes; 27, 136, and 163 in G908R heterozygotes; and 27 and 136 bp in R908R homozygotes.

In order to detect the 1007 fs (Leu1007fsinsC: SNP13, 3020insC) mutation, PCR was carried out using the forward primer 5′- GGCAGAAGCCCTCCTGCAGGGCC-3′ and the reverse primer 5′- CCTCAAAATTCTGCCATTCC-3′ resulting in an amplified fragment of 151 bp in size. After digestion for 16 hours at 37°C with 2 U of ApaI, the following panel was obtained (leucine represents the codon 1007 in the wild-type allele): 151 bp for Leu1007Leu homozygotes; 20, 131, and 151 bp in Leu1007Pro heterozygotes; and 20 and 131 bp in Pro1007Pro homozygotes.

### Detection of *Mycobacterium avium* subsp. *paratuberculosis*

IS900 nested PCR was performed as described previously [12]. Briefly, first round PCR was performed with genomic DNA and primers P90 (5’-GTT CGG GGC CGT CGC TTA GG-3’) and P91 (5’-GAG GTC GAT CGC CCA CGT GA-3’) generating a 398 PCR fragment. In the second round, PCR products from the first round were used as DNA template with primers AV1 (5’-ATG TGG TTG CTG TGT TGG ATG G-3’) and AV2 (5’-CCGCCGCAATCAACTCCAG-3’). The final amplification product was 298 bp long. MAP DNA (ATCC 19698) was used as positive control and run along with the samples.

### Amplicon verification

The identity of the amplicons in all cases was confirmed on samples from two positive healthy controls and 2 IBD patients. For MAP verification the same PCR described for detection ending in a 298 bp amplicon was performed. To confirm the C/T_−13910_ SNP genotyping, primers LACT1-for (5’-GCA TAA AGA CGT AAG TT-3’) and LACT1-rev 5’-ATA TGT TTA CGT TGG ATT CC-3’)[54] were used to generate the amplicon (155 bp). Finally, for NOD2 confirmation the same PCRs described in the genotyping was performed for all three SNPs.

In all cases, bands were excised, extracted and purified (GFX PCR DNA and Gel Band purification kit. Amersham Biosciences, Buckinghamshire, UK). Electrophoresis was performed using an ABI 3130 Genetic analyzer (Applied Biosystems) and base calling was done by Sequencing Analysis 5.2 Software (Applied Biosystems). The obtained sequences were submitted to alignment analyses.

### Statistical analysis

Frequency differences and Hardy-Weinberg equilibrium for the distribution of genotypes among the different groups were tested with chi-squared test. Multiple logistic regression analyses were run for CD versus healthy controls, UC versus healthy controls and IBD (CD and UC combined) versus healthy controls in order to assess the relationship between genotypes and risk of disease. Adjusted values were estimated with 95% confidence limits (CI). P values below 0.05 were considered significant. All statistical analyses were performed using the SAS statiscal package (SAS Insititute Inc., Cary, NC, USA).

## Abbreviations

MAP, Mycobacterium Avium subsp. Paratuberculosis; CD, Crohn’s Disease; UC, Ulcerative Colitis; IBD, Inflammatory Bowel Disease; HC, Healthy Control; LNP, Lactase Non-Persistence; LP, Lactase Persistence.

## Competing interests

The authors subscribe that they have no competing interests related to the present work.

## Authors’ contributions

RJ, LR, AI and JG designed the study; NE, SC and EM performed the experiments; NE and RJ analyzed the data, NE and RJ drafted the paper. All authors critically revised the paper and approved the final version. All authors read and approved the final manuscript.
